# Oligomerization of Family B GPCRs: Exploration in Inter-Family Oligomer Formation

**DOI:** 10.3389/fendo.2015.00010

**Published:** 2015-02-02

**Authors:** Hans K. H. Ng, Billy K. C. Chow

**Affiliations:** ^1^Department of Endocrinology, School of Biological Sciences, The University of Hong Kong, Hong Kong, China

**Keywords:** GPCR, family B, GPCR oligomerization, homomer, heteromer

## Abstract

G-protein-coupled receptors (GPCRs) are classified into A to F subfamilies in which only families A, B, and C are present in mammals. Some of these GPCRs were found to form higher ordered structures such as oligomers with the discovery of interacting receptors in the form of homomers or heteromers. The importance of these oligomers on regulating receptor functions has recently been an intense research focus. It has been proposed that receptor oligomer formation has impact on its physiological importance on receptor trafficking, signaling, ligand-related regulation, and also is related to certain diseases. The present body of knowledge, however, comprises mainly intra-family oligomers formation and their consequences. Inter-family oligomers are recognized but there is limited information. This article aims to provide a current view regarding inter-family GPCR oligomerization in the subfamilies A, B, and C found in mammals.

## Introduction

The vast structural and functional diversity of G-protein-coupled receptors (GPCRs) makes it the largest membrane receptor family. Members of the GPCR family include receptors responding to hormones, neurotransmitters, lipids, photons, ions, nucleotides, among others (1). It has been 20 years since GPCRs are classified according to the A to F system by Kolakowski in 1994. Under this system, only families A, B, and C are found in mammals (2). For these three families of receptors, rhodopsin-like receptors are classified as family A. Family B is further divided into three subfamilies by Harmar into subfamily B1 (secretin-like receptors), B2 (adhesion family), and B3 (Methuselah-like receptors) (3). Family C comprises members having characteristic long N- and C-termini and are responsible for sensing metabotropic glutamate, Ca^2+^ ion, and γ-aminobutyric acid (GABA) (4, 5). The concept of GPCRs oligomerization can be dated back to 1982 when Fraser and Venter discovered that β_2_-adrenergic receptors (β_2_-AR) form dimers in the cell membrane. Their studies provided the first piece of biophysical evidence of GPCR oligomerization using immunoaffinity chromatography, sodium dodecyl sulfate-polyacrylamide gel electrophoresis (SDS-PAGE), and radiation inactivation techniques (6). Since then, receptor dimerization events were demonstrated by co-immunoprecipitation (co-ip) and resonance energy transfer approaches. Among the first studied cases of GPCRs oligomerization, most effort had been focused on family A GPCRs, while oligomerization in family B GPCRs was largely neglected until Miller’s and Schelshorn’s groups provided information on such events in the past decade. The Miller’s group described the homo-oligomerization of secretin receptor (SCTR) (7, 8) and the heteromer formation of SCTR with other family B GPCRs (8, 9), while Schelshorn’s group described glucagon (GCG), glucagon-like peptide-1 (GLP1), glucagon-like peptide-2 (GLP2), and gastric inhibitory polypeptide (GIP) receptors oligomerization (10–12). Apart from providing biophysical proof of GPCRs interactions, physiological consequences of such interaction were also elucidated in the past 15 years when it was gradually unveiled that oligomerization of receptors plays roles in trafficking, ligand-promoted regulation, ligand binding, allostery, as well as signal transduction (13). There also exist *in vivo* evidence that dimerization of family B SCTR with family A angiotensin II receptor type 1a (AT1aR) modulates the signaling properties of the receptors (14). In view of the emerging emphasis on inter-family GPCRs oligomerization and the functional importance of such event, this article will review the basis of GPCRs oligomerization emphasizing on family B GPCRs.

## GPCR Oligomerization

G-protein-coupled receptors were originally thought to function as monomeric molecules, with a 1:1 stoichiometry for one receptor protein to interact with one heterotrimeric G protein (13). However, with the advancement in receptor biology research, GPCR homo- and/or hetero-oligomerization is now generally accepted as a common phenomenon. Although the exact stoichiometry of such interaction remains mostly undetermined under the current limitation of technical difficulties, oligomers of GPCRs have proved to involve in vital roles in terms of functioning of the receptors.

For family A GPCRs, evidences have suggested dimerization as a prerequisite for correct trafficking as well as signaling of certain receptors. Examples are somatostatin receptor and adrenergic receptors (15, 16). Ligand may also regulate the oligomeric state of receptors on the cell surface, either positively for the luteinizing hormone receptor (17) or negatively for the thyrotropin receptor (18). Ligand binding cooperativity was observed to be altered in M2 muscarinic receptor dimers (19).

For family C receptors, receptor oligomerization is essential to the proper functioning of the protein. A classic example is the GABA_B_ receptor (GABA_B_R), which is the heterodimer of the protomers GABA_B_1R and GABA_B_2R. The ligand binding site is located on GABA_B_1R but transportation to the cell membrane as well as G-protein coupling for proper functioning of the receptor can only be achieved when GABA_B_2R is present and heterodimerizes with GABA_B_1R (20, 21). The significance of family B receptors oligomerization will be discussed later in this article.

## Methods to Study Oligomerization of GPCRs

Traditionally, co-ip was used to study receptor oligomers. Observations of oligomer formation in GPCRs primarily rest upon on this technique since the first report by Hebert et al. (22) describing β2-AR receptor dimers (22). However, due to the technical difficulties, methods utilizing the resonance energy transfer between receptors tagged with luminescent or fluorescent proteins have become more popular. In one of the methods, known as bioluminescent resonance energy transfer (BRET), one of the receptor is tagged usually at the C-terminus with the enzyme *Renilla* luciferase (Rlu), which acts as the donor molecule, while the acceptor receptor is tagged with a yellow or green fluorescent protein (YFP/GFP). The two receptor constructs are co-expressed *in vitro*, usually in the form of a saturation experiment when a constant amount of the donor molecule is co-transfected with an increasing amount of acceptor receptors. Upon Rlu activation by its specific substrate, light energy from the reaction can be transferred and excite the acceptor fluorescent protein when the two receptors are in a proximity of <10 nm apart. This fluorescence of the acceptor emission can then be quantified as an indicator of receptor interactions known as the BRET signal. For specific interaction, the BRET signal will increase with increasing concentration of acceptor receptor, indicated by a saturation curve. Negative control is provided as a linear straight line when the BRET signal is produced by the random collision of tagged receptors. The advantages of using BRET methods over co-ip is that BRET improves resolution and more importantly, enable faster screening of dimerization partners (23, 24). Another similar technique replaces the Rlu with a cyan fluorescent protein (CFP), and requires the activation of CFP by laser for resonance energy transfer. This method is known as FRET and eliminates the use of substrate and is primarily useful in applications to study receptor trafficking under the microscope. However, it suffers the disadvantage of having to employ linear unmixing algorithms in the microscope software due to the possible crosstalk of fluorescent signals from the two fluorescent protein tags (25). An improvement to this situation relies on the use of long-lived rare-earth lanthanides energy donors, such as europium. This time-resolved FRET technique lowers the background signal and hence a higher signal:noise ratio can be obtained over normal FRET procedures (26). Other fluorescent based technologies include bimolecular fluorescence complementation and bimolecular luminescence complementation. These experiments use receptors tagged with a part of the fluorescent/luminescent protein that, upon receptor oligomerization, can reassemble into functional protein again (27). Although there exist different approaches to probe for receptor oligomers, most experimental evidence of the physiological consequences of these GPCRs interactions were only found in the *in vitro* model. *In vivo* evidence was poorly lacking until the demonstration of the phenotypes of luteinizing hormone receptor transgenic mice in 2010 (17), and water homeostasis effect of angiotensin II (ANGII) and SCTR homomers and heteromers in 2014 (14).

## Oligomerization of Family B GPCRs and Its Functional Significance

Among the family B GPCRs, the SCTR is the first member of the family cloned from rat (28) and thus is the most comprehensively studied. Using primarily BRET technique, the Miller group provided evidences on SCTR homodimerization (8, 29, 30), as well as heterodimer formation with glucagon-like peptide-1 receptor (GLP1R), glucagon-like peptide-2 receptor (GLP2R), vasoactive intestinal peptide receptors 1 and 2 (VPAC1R, VPAC2R), growth hormone-releasing hormone receptor (GHRHR), parathyroid hormone 1 receptor (PTHR1), parathyroid hormone 2 receptor (PTHR2), and calcitonin receptor-like receptor (CRLR) (8, 9). The specific sites of interaction of SCTR homodimer were the transmembrane 4 of the receptor in which Gly243 and Ile247 residues play a key role (29).

For other members of the family, intra-family homomers includes GLP1R, GLP2R, gastric inhibitory polypeptide receptor (GIPR), glucagon receptor (GCGR) (10–12), PTHR1 (31), VPAC1R, VPAC2R (8), GHRHR (32), calcitonin receptor (CALCR) (33), CRLR (34), corticotrophin-releasing hormone receptor 1 (CRHR1) (35, 36), and pituitary adenylate cyclase-activating polypeptide type I receptor (PAC1) (37), while intra-family heteromers include VPAC1R/VPAC2R (8), GLP1R/GIPR, GLP1R/GCGR, GLP1R/GLP2R, GCGR/GIPR, GCGR/GLP2R, and GIPR/GLP2R (10–12).

For the family B receptors, physiological relevance of dimerization was mainly demonstrated by *in vitro* experiments. First being the dominant negative effect of a mis-spliced SCTR on wildtype SCTR, leading to gastrinoma development (7). The importance of dimerization for receptor trafficking is observed both in SCTR and VPAC1R in which non-dimerizing receptor constructs failed to reach the cell surface (8, 29, 30).

Although no effect was observed for SCTR homodimers (8), ligand binding can negatively affect dimer formation of VPAC1R/VPAC2R/PTHR1 homo/heterodimers (8, 9, 31). Interestingly, GLP1 can positively affect dimer formation for GLP1R and GIPR dimer, while GIP has inhibitory effect on the heterocomplex formation (12, 38). In addition to ligand affecting dimer formation, formation of dimer has an effect on ligand binding as well. Using transmembrane domain 4 (TM4) peptide to disrupt GLP1R homodimer formation, binding of the ligand GLP1 (7–36)-NH2 can be nullified (39). Truncated GHRHR can also lead to conformational change in the dimer complex, which is responsible for the inhibition of GHRH ligand binding (32).

Most family B GPCRs elicit their functions through the cAMP and/or phospholipase C (PLC) signaling cascade. The effect of oligomerization on the cellular mobilization of cAMP and calcium was also one of the research areas in GPCR oligomerization studies. In 2007, it was found that by disrupting SCTR homodimer formation using TM4 peptide, cAMP production is reduced (29). GLP1R and GIPR dimer is also known to decrease the maximal responses of GLP1R in terms of β-arrestin recruitment and calcium mobilization (38, 39). Taken together, oligomerization of GPCRs plays vitally important roles on multiple aspects of cell physiology. Table 1 outlines the intra-family oligomerization of family B GPCRs, the techniques involved and the physiological significance included.

**Table 1:** **Oligomerization of intra-family family B GPCRs**.

Oligomer	Technique	Physiological significance	Reference
**HOMOMERS**			
SCTR/SCTR	BRET/FRET	Relation with gastrinoma; promotes cAMP response	Ding et al. (7), Harikumar et al. (8, 29)
GLP1R/GLP1R	BRET	Promotes ligand binding	Orgaard (10), Roed (11), Schelshorn et al. (12), Harikumar et al. (39)
GLP2R/GLP2R	BRET	N/A	Schelshorn et al. (12)
GIPR/GIPR	BRET	N/A	Schelshorn et al. (12)
GCGR/GCGR	BRET	N/A	Orgaard (10), Roed (11), Schelshorn et al. (12)
PTHR1/PTHR1	BRET/FRET	Ligand reduces oligomerization	Pioszak et al. (31)
VPAC1R/VPAC1R	Co-ip/BRET/FRET	Ligand reduces oligomerization	Harikumar et al. (8)
VPAC2R/VPAC2R	BRET/FRET	Ligand reduces oligomerization	Harikumar et al. (8)
GHRHR/GHRHR	Co-ip	Promotes ligand binding	McElvaine and Mayo (32)
CALCR/CALCR	Co-ip/BRET/FRET	Alters receptor trafficking	Seck et al. (33)
CRLR/CRLR	Co-ip/BRET/FRET	N/A	Heroux et al. (34)
CRHR1/CRHR1	FRET/BRET	N/A	Kraetke et al. (35), Young et al. (36)
PAC1/PAC1	Time-resolved FRET	N/A	Maurel et al. (37)
**HETEROMERS**			
SCTR/GLP1R	BRET	N/A	Harikumar et al. (9)
SCTR/GLP2R	BRET	N/A	Harikumar et al. (9)
SCTR/VPAC1R	FRET/BRET	Receptor trafficking	Harikumar et al. (8, 9, 29)
SCTR/VPAC2R	FRET/BRET	Receptor trafficking	Harikumar et al. (8, 9, 29)
SCTR/GHRHR	BRET	N/A	Harikumar et al. (9)
SCTR/PTHR1	BRET	Ligands reduce oligomerization	Harikumar et al. (9)
SCTR/PTHR2	BRET	Ligands reduce oligomerization	Harikumar et al. (9)
SCTR/CRLR	BRET	N/A	Harikumar et al. (9)
GCGR/GLP1R	BRET	N/A	Orgaard (10), Roed (11), Schelshorn et al. (12, 38)
GCGR/GLP2R	BRET	None	Schelshorn et al. (12, 38)
GCGR/GIPR	BRET	None	Schelshorn et al. (12, 38)
GIPR/GLP1R	BRET	GLP-1 induces oligomerization and flattened Ca^2+^ response; GIP reduces oligomerization but does not alter Ca^2+^ response	Harikumar et al. (39), Schelshorn et al. (12, 38)
GIPR/GLP2R	BRET	None	Schelshorn et al. (12, 38)
GLP1R/GLP2R	BRET	N/A	Orgaard (10), Roed (11), Schelshorn et al. (12, 38)
VPAC1R/VPAC2R	Co-ip/BRET	Ligand reduces oligomerization	Harikumar et al. (8)

*Technique involved and physiological significance are described; N/A, information not available*.

## Inter-Family GPCRs Oligomerization

Since the maturation of the concept of GPCR oligomerization, its functional implications are gradually understood and appreciated. Experimental evidences on oligomer formation as well as physiological importance has been accumulating first in families A and C, then in family B members in the past years. However, most studies so far has been focusing on intra-family receptor oligomerization, inter-family events are poorly understood. The fact that there exist little sequence homology between families A, B, and C receptors, despite having shared common general morphology, may explain this scarcity of information (5, 40). Up till now, documented inter-family events include family B GIPR with family A members β_2_-AR and opsin. In their experiment, they use the BRET method in a heterologous expression system to discover that cAMP production is increased upon ligand stimulation compared to monomers (41). Family B CRHR1 with family A vasopressin receptor 1b (V1bR) was studied again using the BRET technique but instead of tagging the receptors at the C-termini, they are tagged at the N-termini (36). Family B GCGR with family A cholecystokinin A receptor was also reported (CCKAR) (10). Among these, family B SCTR with family A AT1aR shows the most convincing evidence. Based on the observation that both drinking behavior and vasopressin expression and release are impaired in secretin (SCT) or SCTR knockout mice upon intracerebroventricular (i.c.v.) injection of ANGII (42), it was deduced that a SCT/SCTR axis is essential to the proper functioning of ANGII in the central nervous system. With the coexpression of AT1aR and SCTR in the paraventricular nucleus, receptor heterocomplex formation was hypothesized to be the reason behind this observation. By using BRET techniques, it was found that SCTR interacts with AT1aR specifically, but not angiotensin II receptor type 2 (AT2R). When the receptors were expressed together *in vitro* and stimulated with SCT alone, cAMP response was blunted compared to cells bearing only SCTR or SCTR with the non-dimerizing AT2R. However, cAMP production could be restored when ANGII was also present or when SCTR was co-expressed with a constitutively active mutant AT1aR, but not with the ANGII binding-deficient mutant AT1aR-K199A. Together with the fact that AT1aR cannot stimulate the cAMP signaling pathway, it was concluded that an active conformation of the AT1aR was responsible for regulating SCTR in mediating cAMP responses. In line with previous finding (29), the role of TM peptides on heterocomplex formation was also elucidated. It was found that peptides derived from SCTR’s TM 2 and 4 (STM-II/IV), and AT1aR’s TM 1 and 4 (ATM-1/4), could inhibit heteromer formation, while only STM-IV or/ATM-4 peptides could suppress the homomers of SCTR or AT1aR, with alanine mutants of these peptides reversing the situation. ATM-1 was chosen to investigate heteromer-specific actions as this peptide is neither capable of disrupting SCTR nor AT1aR homomer formation. By using these TM peptides as a tool, specifically ATM-1 and its mutant counterpart, it was demonstrated that full activity of SCT-stimulated SCTR requires the activation of AT1aR as a prerequisite. To further investigate the physiological relevance of SCTR/AT1aR heteromer in relation to water homeostasis, i.c.v. injections of the TM peptides and their mutants were administered to mice under hyperosmolality stress. All the four aforementioned TM peptides can suppress drinking behavior of these mice under hyperosmolality to different degrees. Of which, STM-IV, ATM-1, and ATM-4 can inhibit drinking behavior to a level similar to that of SCTR knockout mice or mice injected with the protein kinase A inhibitor H89, for the case of STM-II the effect was not as prominent. These results were reversed when the alanine mutants TM peptides were used instead. The experiments demonstrate that receptor heterocomplex of SCTR and AT1aR plays a role in regulating water drinking behavior *in vivo*. Furthermore, i.c.v. injection of a combination of low doses of both SCT and ANGII produces an effect on drinking behavior comparable to injection of high doses of these hormones alone, which is significantly greater than that when the hormones were given singly at low doses. This synergistic effect of the hormones in the central nervous system hints that the physiological effect of SCTR can be prominently enhanced with ANGII. This important study strongly suggests the significance of inter-family heterodimer formation on physiology (14). Table 2 summaries the heteromer formation of inter-family GPCRs.

**Table 2:** **Oligomerization of inter-family family B GPCRs**.

Heteromers	Technique	Physiological significance	Reference
GIPR/β2-AR	BRET	Potentiates cAMP responses	Vrecl et al. (41)
GIPR/opsin	BRET	Potentiates cAMP responses	Vrecl et al. (41)
CRHR1/V1bR	BRET	N/A	Young et al. (36)
GCGR/CCKAR	BRET	N/A	Orgaard (10)
SCTR/AT1aR	BRET/FRET	Reduces secretin’s cAMP response; alters drinking behavior	Lee et al. (14)

*Technique involved and physiological significance are described; N/A, information not available*.

## Conclusion and Future Perspective

Since there is a growing body of evidences on the functional importance of GPCR oligomerization but information on potential oligomerizing partners within families A, B, and C is lacking; the area of GPCRs oligomerization within these families is being explored in our laboratory recently. Initial approach includes setting up a GPCR–YFP library with GPCRs tagged with YFP. Using this scheme, any GPCR having tagged with Rlu can be screened against this library for oligomer partners. Not only can this study compensate our current paucity of information on inter-family GPCR oligomers but also the cellular co-localization of these partners and their physiological relevance can be elucidated. If the receptors are found to be co-localized and serving similar functions, further biochemical analysis can be made to assay for the functional significance of the receptor interaction. As such, a number of novel mechanisms in controlling cellular activities may be discovered. Such discoveries could in turn facilitate the development of biochemical tools for scientific research or *in vitro* diagnostics. As around 30–40% of the pharmaceutical drugs at present are targeting GPCRs, this basic research on the role of GPCR oligomerization may pave the path for developing pharmaceutical precursors, which may eventually become the answers to a variety of diseases.

## Conflict of Interest Statement

The authors declare that the research was conducted in the absence of any commercial or financial relationships that could be construed as a potential conflict of interest.
